# Feasibility of utilizing the Modified Centor Criteria in adult virtual care

**DOI:** 10.1017/S1463423625000052

**Published:** 2025-02-26

**Authors:** Ahmed Allabban, Samay Shah, Neal Sikka, Colton Hood

**Affiliations:** 1 Department of Emergency Medicine, George Washington University, Washington, DC, USA; 2 Department of Emergency Medicine, King Abdulaziz University Faculty of Medicine, Jeddah, Saudi Arabia

**Keywords:** clinical decision rule, Modified Centor Criteria, strep test, streptococcal pharyngitis, telemedicine

## Abstract

The Modified Centor criteria (MCC) is a validated clinical decision tool determining the need for testing in suspected *Streptococcal pharyngitis*. This study aims to understand the use of this tool to guide testing during remote evaluation. Patients with sore throats and no more than 3 days of symptoms were recruited from the emergency department and urgent care at an urban academic centre in 2019–2022. All patients enrolled were 18 years or older. Each participant had three MCC recorded, once in person and again by two different blinded telemedicine providers (TP). A total of 172 patients were screened and 40 were enrolled, they had a mean age 32 and were 43% male. We calculated inter-rater reliability between in-person and telemedicine providers, using a threshold score of strep testing (≥2) and non-testing scores (<2). Cohen’s kappa between in-person and telemedicine providers was 0.68 while the TP were in complete agreement.

## Introduction

Telehealth utilization increased tremendously during the COVID-19 pandemic. A study by the Assistant Secretary for Planning and Evaluation found that there were 52.7 million Medicare visits conducted via telehealth in 2020, a 63-fold increase compared to the previous year (Samson *et al*., 2021). This growth slowed in 2021, but telehealth utilization was still considerably higher than it was in 2019 (Lo *et al*., 2022).

Telehealth utilization will likely reach a steady percentage of all visits based on a number of provider and community factors, including improved clinician attitudes towards telehealth, increased investment into digital health, and certain regulatory changes that have allowed for the expanded use of telehealth (Bestsennyy *et al*., 2021). Additionally, patients appear to have a favourable view towards this modality – in the Telehealth Impact Study conducted in 2021, over 2,000 English-speaking respondents from across the US reported very high satisfaction with care, and the majority stated that they plan to continue using telehealth in the future (Campion *et al*., 2021).

Given more frequent care delivery through virtual modalities, it is important to understand whether in-person patient evaluation strategies can be effectively utilized in the virtual environment. Clinical decision rules (CDR) are used in practice today to assist in diagnosis and treatment decisions. They are built from clinical data and incorporate elements of patient history, symptoms, vital signs, and physical exam findings to guide next steps in management – including the likelihood that a patient has a particular condition or whether further diagnostic testing is warranted. There has been little investigation into whether CDRs used in traditional in-person visits are valid when utilized during virtual visits (Ebell *et al*., 2021). It is important to determine a CDR’s validity and consider appropriate modifications and thresholds for use that optimize its performance to ensure quality care is provided when applied to a virtual setting.

We investigated the Modified Centor Criteria (MCC), a validated clinical decision rule used to determine the probability that a patient’s pharyngitis is due to Group A beta-haemolytic streptococcal (GABHS) infection (McIsaac *et al*., 1998; McIsaac *et al*., 2004; Fine *et al*., 2012). Bacterial streptococcal infection is one of the most common reasons for ambulatory care visits in the United States every year, and early detection is critical, as it can help improve outcomes and avoid serious complications like rheumatic fever (Arnold and Nizet, 2018). Additionally, effective virtual management of patients with communicable illnesses (i.e. limiting in-person follow-up to only when it is truly necessary) can reduce the spread of infection. The MCC incorporates five elements: age, fever, cough, swollen/tender anterior cervical lymph nodes, and tonsillar exudate or swelling (Table 1).

**Table 1.** Elements of the Modified Centor Criteria

Criteria	Points
Temperature >38 ºC	1
No cough	1
Tonsillar swelling exudate	1
Tender anterior cervical lymphadenopathy	1
Age (years)	
3–14	1
15–44	0
≥ 45	−1

In this decision aid, a score of less than two indicates that the risk for (GABHS) pharyngitis is <11 % and testing is not recommended (Choby, 2009). Most of the elements of this CDR are history-based. The two criteria that are challenging to ascertain virtually are cervical lymphadenopathy, which requires examination by palpation, and tonsillar exudate or swelling, which requires examination through visualization. In this study, we aimed to determine the inter-rater reliability of MCC scores obtained by in-person providers compared with those obtained by telemedicine providers. We hypothesized that there would be no significant difference between the two sets of scores.

## Methods

### Study design

This was quasi-experimental study of data collected from a convenience sample of emergency department (ED) and outpatient clinic patients who presented with a chief complaint of a sore throat or similar symptoms from December 2019 to March 2022. The majority of patients were enrolled prior to March 2020. Enrolment was then stopped due to the COVID-19 pandemic until the spring of 2021. The study obtained Institutional Review Board approval at George Washington University, where all study participants provided informed consent for the study. The study was designed, developed, and completed as part of a Telemedicine and Digital Health Fellowship at George Washington University.

### Setting and population

This was a single-centre study executed in Washington, DC, United States, at an academic medical centre with approximately 74,000 annual ED patient visits and a busy outpatient practice. Patients were included in the study if they were aged seven or above with the chief complaint of sore throat or throat pain (including difficulty swallowing, scratchy throat, or painful swallowing), had an Emergency Severity Index (ESI) of 2, 3, 4, or 5, their symptoms onset occurred within the past three days, and have not been assessed previously by a medical provider. Patients were excluded if they did not provide informed consent, were unable to understand consent (i.e. influence of alcohol or drugs, cognitively impaired), were having an acute behavioural health exacerbation, were visually impaired, deaf, or hard of hearing, were prisoners, or had an ESI score of 1, and were non-English speakers.

### Data processing and outcomes

Trained research assistants (RAs) identified potential participants based on their listed chief complaint on the ED electronic health record (EHR) tracking board or in outpatient clinic waiting rooms. RAs were trained as clinical presenters in the MCC. The RAs’ training focused on accurately palpating the anterior cervical lymph nodes and identifying lymph node enlargement; the other focus of the training was on obtaining the optimal view to visualize and capture a still photo and video of the throat. RAs approached eligible patients for enrolment. Eligible and consented patients (by the patient or their parent, when under 18) were evaluated asynchronously by two telemedicine providers and in-person physicians for MCC score calculation. RAs recorded all elements of the MCC except the question about the presence of tonsillar swelling or exudate. The telemedicine providers assessed the posterior pharynx and tonsils by reviewing the images and video recordings that the RAs collected of the back of the throat for each enrolled patient and uploaded to a secure cloud storage. The participant also completed a routine evaluation by the in-person provider, and the MCC score was recorded. The in-person clinical evaluator was blinded to the clinical presenter and telemedicine provider evaluation. The two telemedicine providers were blinded to each other’s evaluation.

The study’s primary outcome was to compare the MCC score of ≥2 or <2 between the in-person emergency medicine providers and telemedicine clinicians assisted by trained clinical presenters. The secondary outcome was to compare the MCC score of ≥2 or <2 between the two telemedicine providers. We also examined a higher MCC score threshold of ≥3 as well as inter-rater reliability in examining photographs of the throat for exudate asynchronously between two telemedicine providers.

### Data analysis

Each subject with a strep test result was classified into a testing or non-testing category based on their MCC score. Patients with an MCC score of ≥2 were labelled as testing, and patients with a score of <2 were labelled as non-testing. For each participant, three providers’ evaluations were completed (one in-person and two telemedicine). Inter-rater reliability was determined using Cohen’s kappa coefficient calculated between the in-person physician and the two telemedicine providers. A similar analysis was completed between the two telemedicine providers, as well as for exudate only, and at the high MCC score threshold of ≥3. Cohen’s kappa and confidence intervals were calculated using Stata 15 (Stata Corp, College Station, TX) with confidence intervals calculated using KAPUTIL package (Harrison, 2004).

## Results

**Figure 1.**
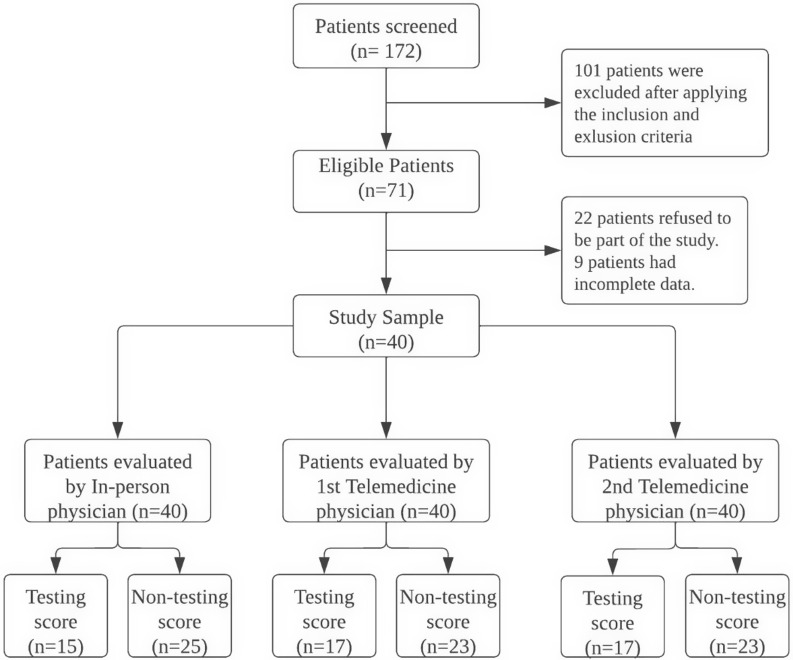
Flow chart of patients screened and enrolled in the study.

During our study period, 172 patients were screened for enrolment, and 40 patients were enrolled and provided complete data. Participants had an average age of 32, and 43% were male. All enrolled patients were 18 years or older. In-person (IP) provider evaluations assigned testing MCC scores (≥2) for 15 patients and non-testing (<2) scores for 25 patients. The first and second telemedicine (TM1, TM2) providers assigned identical testing and non-testing MCC scores (recommend testing in 17 patients, no testing in 23 patients, see Figure 1).

We observed agreement in testing and non-testing between the IP, TM1, and TM2 providers in 34 patients (85%) and disagreement in 6 patients (15%). Among the six patients with disagreements, two patients (5%) were observed to be under-triaged by both telemedicine providers, and four patients (10%) were under-triaged by the in-person providers. The result of Cohen’s kappa for inter-rater reliability between in-person and both telemedicine providers using a threshold of MCC ≥2 for testing and <2 for non-testing was 0.68 (95% CI 0.39–0.85). The result of Cohen’s kappa for inter-rater reliability between TM1 and TM 2 in testing for a threshold ≥ 2 and non-testing was 1.0 (95% CI 0.82–1.0).

When looking at a MCC score threshold of testing of ≥ 3, Cohen’s kappa for inter-rater reliability between the in-person and TM1 in the evaluation of exudate and MCC score ≥ 3 was 0.45 (95% CI 0.13–0.69) and 0.54 (95% CI 0.15–0.81), respectively, and between the in-person and TM2 was 0.45 (95% CI 0.13–0.69) and 0.47 (95% CI 0.1–0.76). The comparison between TM1 and TM2 in evaluating the exudate only and MCC score ≥ 3 was 0.73 (95% CI 0.41–0.89) and 0.68 (95% CI 0.28–0.88), respectively (see Table 2). The RA collected images of the throat and evaluations of lymph nodes altered the MCC score in 9 (22.5%) and 7 (17.5%) patients, respectively (22.5%).

**Table 2.** Cohen’s kappa values comparing Modified Centor Scores and exudate evaluations between the in-person provider (*IP*), telemedicine provider 1 (TM1), and telemedicine provider 2 (TM2)

	TM1: TM2	TM1: IP	TM2: IP
Evaluation of throat for exudate only	0.73 (95% CI 0.41–0.89)	0.45 (95% CI 0.13–0.69)	0.45 (95% CI 0.13–0.69)
Modified Centor Score of ≥ 2	1.0 (95% CI 0.82–1.0)	0.68 (95% CI 0.39–0.85)	0.68 (95% CI 0.39–0.85)
Modified Centor Score ≥ 3	0.68 (95% CI 0.28–0.88)	0.54 (95% CI 0.15–0.81)	0.47 (95% CI 0.1–0.76)

We described the pattern of findings regarding the presence of exudate and the MCC score between in-person providers, TM1, and TM2 among the five patients with a positive rapid strep test (see Table 2). Using the MCC value of ≥2 for testing, two patients with a positive strep test would not have been recommended for testing by TM1, TM2, or in-person.

## Discussion

We found that there was a higher degree of agreement between in-person providers and telemedicine providers in calculating the Modified Centor score with a threshold testing score of ≥ 2. As care delivery models evolve, we must determine whether standards of in-person care can be applied. According to Choby, a score of 2 represents 11–17% risk of (GABHS) infection, and score of 3 represents a 28–35% risk of GABHS infection (Choby, 2009). The MCC is impacted by two factors that are not history-related, the presence of anterior cervical lymphadenopathy and exudate. In our study, the images of the throat and evaluations of lymph nodes made by trained RAs did alter the MCC score in some cases. Not capturing the presence of lymphadenopathy and not identifying exudate via a still image or video evaluation has the potential to change the score by 2 points. When we moved our threshold for testing to 3, we found lower inter-rater reliability. It is important to note that in the 12.5% of patients with a positive strep test, 2 of the 5 were not recommended for testing by either in-person or telemedicine providers. These results show the limits of using the MCC in the evaluation of sore throat in any situation. However, it can still be a useful tool, especially when utilizing a lower testing threshold as the improved inter-rater reliability suggests. Further, our two telemedicine providers, one with limited telehealth experience and one with significant telehealth experience, had an k = 1 for an MCC testing threshold of 2 and k = 0.7 for identifying exudates.

Future studies can expand upon these results and validate the use of telemedicine to determine the MCC score. They should also determine if patients could assess their own lymph nodes or if the exam can be removed all together. Studies could also explore if computer vision could automate the decision rule completely. If it is determined that the MCC can be appropriately used in virtual visits to accurately evaluate the risk of strep infection, it may be possible to limit the rates of in-person follow-up, increasing the convenience of telemedicine and patient satisfaction. Additionally, appropriate triage reducing in-person evaluations may limit the spread of infection.

This study was limited by its small adult-only sample size. Further study would need to take place in paediatric populations. The low enrolment rate is due to the MCC guidelines, as the decision tool cannot be applied if patients have had symptoms for over 3 days. Research assistants helped capture the aspects of the MCC for the purposes of the study. However, in a real-world telemedicine setting, the patient may be alone and must capture the visual elements on their own. Currently, these results may be most applicable to an environment where there are trained clinical presenters. Manoeuvring the camera and lighting in a manner that allows for quality assessment by the physician may prove to be challenging for some patients and to utilize the decision rule in its current state providers may need to be trained on how to guide patients in ways that optimize the assessment. Patients do need to evaluate and report their own assessment of lymph node swelling and a consideration could be made to assume they are present when calculating the score. The presence of lymphadenopathy is reported to have a positive likelihood ratio of 0.47 to 2.9 and a negative likelihood ratio of 0.58 to 0.92 (Choby, 2009).

## Conclusion

As new care models evolve and patients determine the modalities by which they prefer to receive care, it is important to determine whether standards of in-person care can be applied to virtual visits. Clinical decision rules, like the MCC, should be studied further to determine if they are appropriate to use or be adapted for use in telemedicine so that evidence can guide standards for virtual visits.
